# Radiographic union score for hip substantially improves agreement between surgeons and radiologists

**DOI:** 10.1186/1471-2474-14-70

**Published:** 2013-02-25

**Authors:** Mohit Bhandari, Mary M Chiavaras, Naveen Parasu, Hema Choudur, Olufemi Ayeni, Rajesh Chakravertty, Simrit Bains, Alisha Hak, Sheila Sprague, Brad Petrisor

**Affiliations:** 1 McMaster University, 1280 Main Street West, Hamilton, Ontario, L8S 4L8, Canada; 2 University of Toronto, 27 King’s College Circle, Toronto, Ontario, M5S 1A1, Canada; 3 University of Western Ontario, 1151 Richmond Street, London, Ontario, N6A 3K7, Canada

**Keywords:** Hip fractures, Reliability, Fracture healing, Radiographs

## Abstract

**Background:**

Despite the prominence of hip fractures in orthopedic trauma, the assessment of fracture healing using radiographs remains subjective. The variability in the assessment of fracture healing has important implications for both clinical research and patient care. With little existing literature regarding reliable consensus on hip fracture healing, this study was conducted to determine inter-rater reliability between orthopedic surgeons and radiologists on healing assessments using sequential radiographs in patients with hip fractures. Secondary objectives included evaluating a checklist designed to assess hip fracture healing and determining whether agreement improved when reviewers were aware of the timing of the x-rays in relation to the patients’ surgery.

**Methods:**

A panel of six reviewers (three orthopedic surgeons and three radiologists) independently assessed fracture healing using sequential radiographs from 100 patients with femoral neck fractures and 100 patients with intertrochanteric fractures. During their independent review they also completed a previously developed radiographic checklist (Radiographic Union Score for Hip (RUSH)). Inter and intra-rater reliability scores were calculated. Data from the current study was compared to the findings from a previously conducted study where the same reviewers, unaware of the timing of the x-rays, completed the RUSH score.

**Results:**

The agreement between surgeons and radiologists for fracture healing was moderate for “general impression of fracture healing” in both femoral neck (ICC = 0.60, 95% CI: 0.42-0.71) and intertrochanteric fractures (0.50, 95% CI: 0.33-0.62). Using a standardized checklist (RUSH), agreement was almost perfect in both femoral neck (ICC = 0.85, 95% CI: 0.82-0.87) and intertrochanteric fractures (0.88, 95% CI: 0.86-0.90). We also found a high degree of correlation between healing and the total RUSH score using a Receiver Operating Characteristic (ROC) analysis, there was an area under the curve of 0.993 for femoral neck cases and 0.989 for intertrochanteric cases. Agreement within the radiologist group and within the surgeon group did not significantly differ in our analyses. In all cases, radiographs in which the time from surgery was known resulted in higher agreement scores compared to those from the previous study in which reviewers were unaware of the time the radiograph was obtained.

**Conclusions:**

Agreement in hip fracture radiographic healing may be improved with the use of a standardized checklist and appears highly influenced by the timing of the radiograph. These findings should be considered when evaluating patient outcomes and in clinical studies involving patients with hip fractures. Future research initiatives are required to further evaluate the RUSH checklist.

## Background

Hip fractures have high rates of morbidity and mortality [1-3], and are prone to delayed and nonunions [4]. Given the importance of fracture healing on patient outcome in both clinical practice and in guiding clinical research decisions, it is critical to ensure assessments of fracture healing are reliable and valid. The assessment of hip fracture healing is highly subjective and lacks a gold standard, resulting in disagreements in its assessment among orthopaedic surgeons and radiologists [4-9]. There is a wide array of definitions for fracture healing, which aids in the conclusion that there is little consensus among professionals for when a fracture is deemed healed [10]. This lack of consistency renders the comparison of study results with the outcome of fracture healing difficult, as standardization does not exist [10]. As a result there is a need for a standardized system of healing assessment in patients with hip fractures.

The objectives in this study were therefore to: 1) evaluate inter-observer hip fracture healing agreement between surgeons and radiologists, 2) evaluate the performance of a previously developed checklist the Radiographic Union Score for Hip (RUSH) for fracture healing by examining its effect on inter-observer agreement, and 3) determine if agreement improved when using sequential radiographs in comparison to a previous study in which single radiographs with an unknown time from surgery were assessed [11]. We hypothesized improved agreement between surgeons and radiologists when compared to our previous study and improved agreement with the use of the RUSH checklist.

## Methods

### Overview

**Figure 1:**
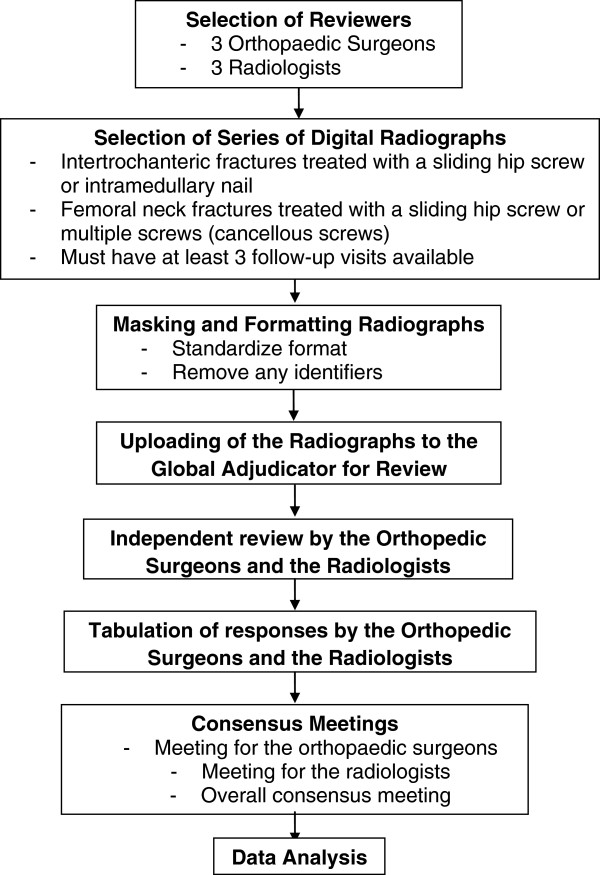
Study procedures: inter-rater reliability assessment.

Prior to initiation, our study was approved by McMaster University / Hamilton Health Sciences Research Ethic Board (REB: 11–169). Briefly, a panel of six reviewers, equally comprised of orthopedic surgeons and radiologists, independently assessed for fracture healing 100 surgically treated femoral neck and 100 intertrochanteric fractures and scored the fractures using a checklist (RUSH). Each case was represented by a series of anteroposterior and cross-table lateral radiographic views of a hip fracture. The radiographs were performed immediately after surgery for a baseline assessment. Each patient had three to five radiographs at various time points within 18 months of their hip fracture. All radiographs were dated, therefore reviewers could determine the time from injury for each follow-up radiograph. Consensus meetings were held to reach agreement within the surgeon and radiologist groups, and then between the reviewer groups. This information was used in the subsequent data analysis. A summary of our methods is presented in Figure 1.

### Development of the radiographic union score in Hip fractures (RUSH) score

The RUSH checklist (Additional file 1: Appendix A) is a novel scoring system for hip fractures. This checklist was developed analogous to the Radiographic Union Score for Tibial fractures (RUST) checklist [12], and was piloted among surgeons and radiologists to ensure early face and content validity. The RUSH checklist was first used in an earlier study we conducted that assessed hip fracture healing agreement using a single radiograph; reviewers were unaware of the time from surgery for each radiograph [11]. It was developed in an effort to standardize hip fracture healing assessment and incorporated several definitions of fracture healing found in the literature, including cortical and trabecular bridging and fracture line disappearance.

### Reviewers

Our panel of reviewers included three musculoskeletal specialized radiologists and three orthopedic surgeons who routinely manage hip fractures. The inclusion of two different medical specializations in the panel allowed us to determine potential differences in the patterns of assessment and also to evaluate the applicability of our checklist to the two specialties most involved in fracture healing assessment. The reviewers were specifically selected for participation based on their experience and training in the assessment and treatment of musculoskeletal trauma, especially hip fractures.

### Selection of cases

Eligible cases of hip fractures had immediate post-operative images and images available for at least three to five subsequent follow-up visits, each consisting of at least two radiographic views. In the case of lateral views, if a cross-table view was not available, an oblique view was obtained. 100 femoral neck and 100 intertrochanteric cases of fractures were selected to reflect the two most common types of hip fractures. We selected series of radiographs that had a single fracture and were treated with a sliding hip screw, intramedullary nailing, or cancellous screws. The reviewers were not involved with the selection of the radiographs.

### Outcome measures

**Figure 2:**
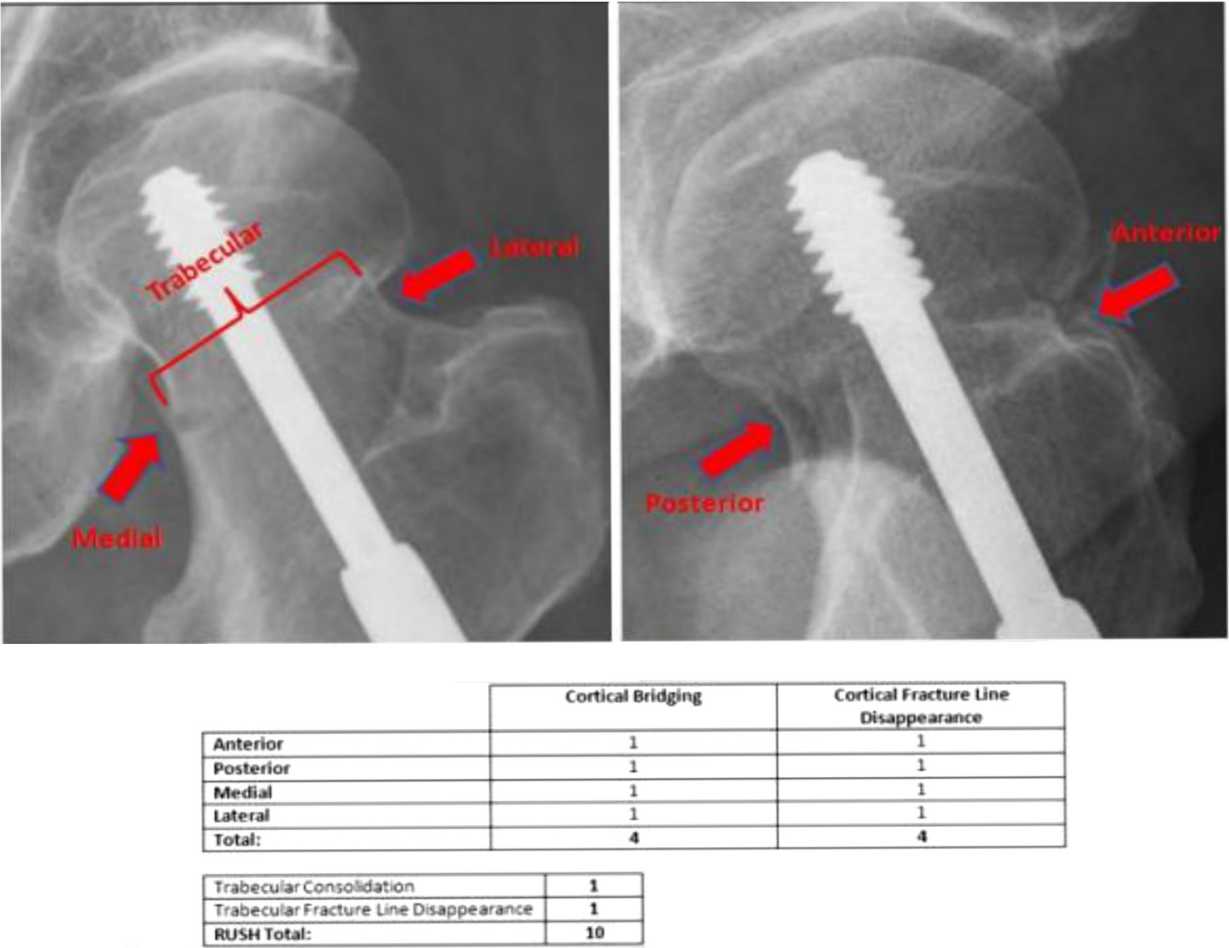
Early post-operative radiographs and RUSH assessment.

**Figure 3:**
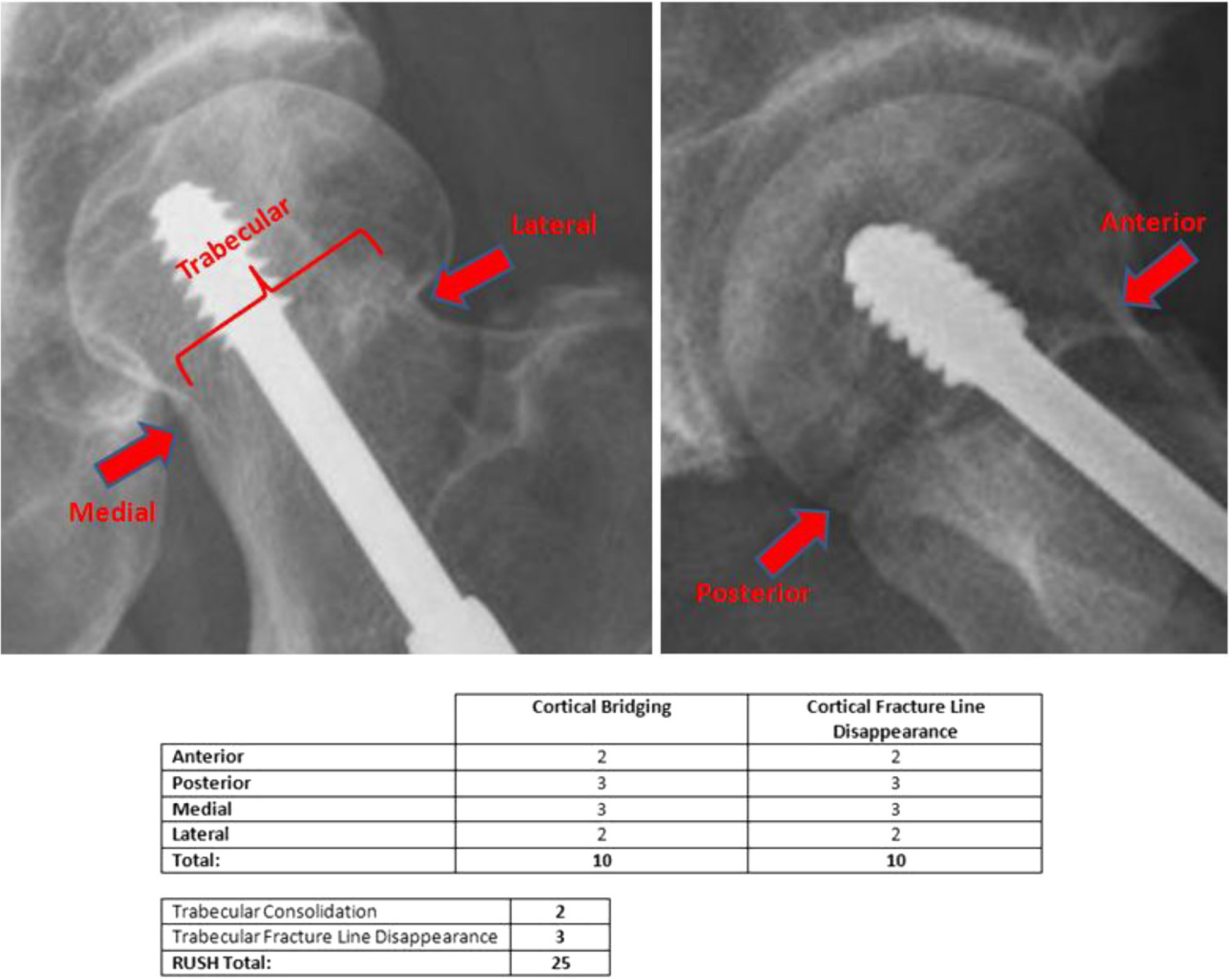
Late post-operative radiographs and RUSH assessment.

Reviewers first assessed whether the fracture was healed (yes or no) based upon their overall assessment of the radiograph using their experience and expertise. After performing this assessment, each reviewer completed the RUSH checklist with specific questions about each of the cortices and trabecular bridging across the fracture. The RUSH checklist is scored by assessing four component scores of cortical bridging, cortical disappearance, trabecular consolidation, and trabecular disappearance. The cortical bridging index score, with a range of 4 to 12, was determined by scoring each of four cortices from 1 to 3. The cortical disappearance score, also with a range of 4 to 12, was determined similarly, except it was based on the visibility of the fracture line at each of the four cortices. Two trabecular indices were scored from 1 to 3 each based on consolidation for one of the indices, and fracture line disappearance for the other. The overall RUSH score therefore ranged from a minimum of 10 to a maximum of 30. Reviewers also assessed the quantity of callus formation and, if applicable, commented on the quality of the radiographs for each case. An example of this assessment is demonstrated in Figures 2 and 3, which show different radiographs from the same patient. Figure 2 displays early post-operative radiographs and the corresponding RUSH score broken down into its components, while Figure 3 shows late radiographs from the same patient and a higher RUSH score.

Results from the overall impression of fracture healing and the score from the RUSH checklist were then compared to the above mentioned study that was completed previously to determine if agreement in the present study was improved [11].

### Adjudication process for fracture healing

100 cases each of femoral neck and intertrochanteric fractures were uploaded for online display on a secured, password protected e-adjudication platform (Global Adjudicator™). Cases contained four to six visits, each with two radiographs. Dates were provided for the radiographs to demonstrate the time from surgery; the first visit for every case contained radiographs obtained immediately after surgery. All reviewers were previously trained and experienced on the use of this system and on the use of the RUSH checklist. Their assessment was entirely independent and the reviewers were unaware of the assessments of their colleagues until the consensus meetings.

After review, their assessments were tabulated and consensus meetings were held to discuss any disagreements on fracture healing and to reach consensus on each case. The radiologists and orthopaedic surgeons initially convened to obtain consensus separately within their groups before meeting to reach an overall consensus (all 6 reviewers). This consensus information was used to determine the inter-observer agreement between groups.

### Sample size

Having all six reviewers rate each radiograph and using binary outcomes (i.e. yes versus no), 100 radiographs will provide a confidence interval around kappa with a width of 0.10.

### Data analysis

Agreement in assessments of fracture healing and overall RUSH score were determined using the intraclass coefficient (ICC) score with 95% confidence intervals. Inter-observer agreement was determined between reviewer groups; that is, the agreement between the consensus answers of the surgeon group and the consensus answers of the radiologist group was determined. This was done separately for each of the two fracture types.

As they are numerically equivalent, the same guidelines for interpretation of kappa values can be applied to the ICC. Landis and Koch suggest that kappa of 0 to 0.2 represents slight agreement, 0.21 to 0.40 fair agreement, 0.41 to 0.60 moderate agreement, and 0.61 to 0.80 substantial agreement [13]. A value above 0.80 is considered almost perfect agreement. These were the guidelines we used in the interpretation of our results. The value of the ICC ranges from +1, in which case there is perfect agreement, to −1, which corresponds to absolute disagreement.

Finally, RUSH scores and healing were correlated with overall assessments of fracture healing.

## Results

### Overall impression of fracture healing

Overall, reviewer agreement between radiologists and orthopedic surgeons for fracture healing assessment was moderate for both femoral neck (ICC = 0.60, 95% CI: 0.42-0.71) and intertrochanteric fractures (0.50, 95% CI: 0.33-0.62). Agreement between radiologists and surgeons increased as the radiographs were taken later after surgery. For femoral neck fractures, agreement increased from fair (ICC = 0.213, 95% CI: 0.061-0.351) for radiographs taken from 0 to 3 months, to moderate (ICC = 0.466, 95% CI: 0.325-0.587) for radiographs taken 6 months or more after surgery. For intertrochanteric fractures the pattern was similar, with agreement increasing from fair, for radiographs taken from 0 to 3 months after surgery (ICC = 0.234, 95% CI: 0.096-0.359) to moderate for those taken after 6 months (ICC = 0.536, 95% CI: 0.268-0.729).

### RUSH checklist

The agreement for the overall RUSH score from the checklist was near perfect between radiologists and orthopedic surgeons with little difference between the femoral neck (ICC = 0.85, 95% CI: 0.82-0.87) and the intertrochanteric fracture (ICC = 0.88, 95% CI: 0.86-0.90) assessments. The agreement for the individual RUSH score components were also high, ranging from substantial to near perfect (Table 1). Agreement between radiologists and surgeons for RUSH scores for femoral neck fracture radiographs taken at 0 to 3 months after surgery was substantial (ICC = 0.709, 95% CI: 0.638 – 0.767), and increased to near perfect for radiographs taken after 6 months (ICC = 0.842, 95% CI: 0.786 – 0.884). For intertrochanteric fractures, agreement was near perfect for radiographs obtained from 0–3 months after surgery (ICC = 0.816, 95% CI: 0.770 – 0.853), but was substantial for radiographs taken after 6 months of surgery (ICC = 0.710, 95% CI: 0.503 – 0.840).

**Table 1:** ICC scores for agreement between surgeons and radiologists on rush component scores (95% confidence interval)

**Parameter**	**Femoral neck**	**Intertrochanteric**
**Overall RUSH score**	0.85	0.88
	(0.82-0.87)	(0.86-0.90)
**Cortical bridging**	0.79	0.88
	(0.69-0.85)	(0.86-0.90)
**Cortical disappearance**	0.78	0.85
	(0.74-0.81)	(0.82-0.88)
**Trabecular consolidation**	0.72	0.74
	(0.67-0.77)	(0.69-0.78)
**Trabecular disappearance**	0.59	0.69
	(0.50-0.67)	(0.63-0.73)

### Comparison of agreement to initial study

**Figure 4:**
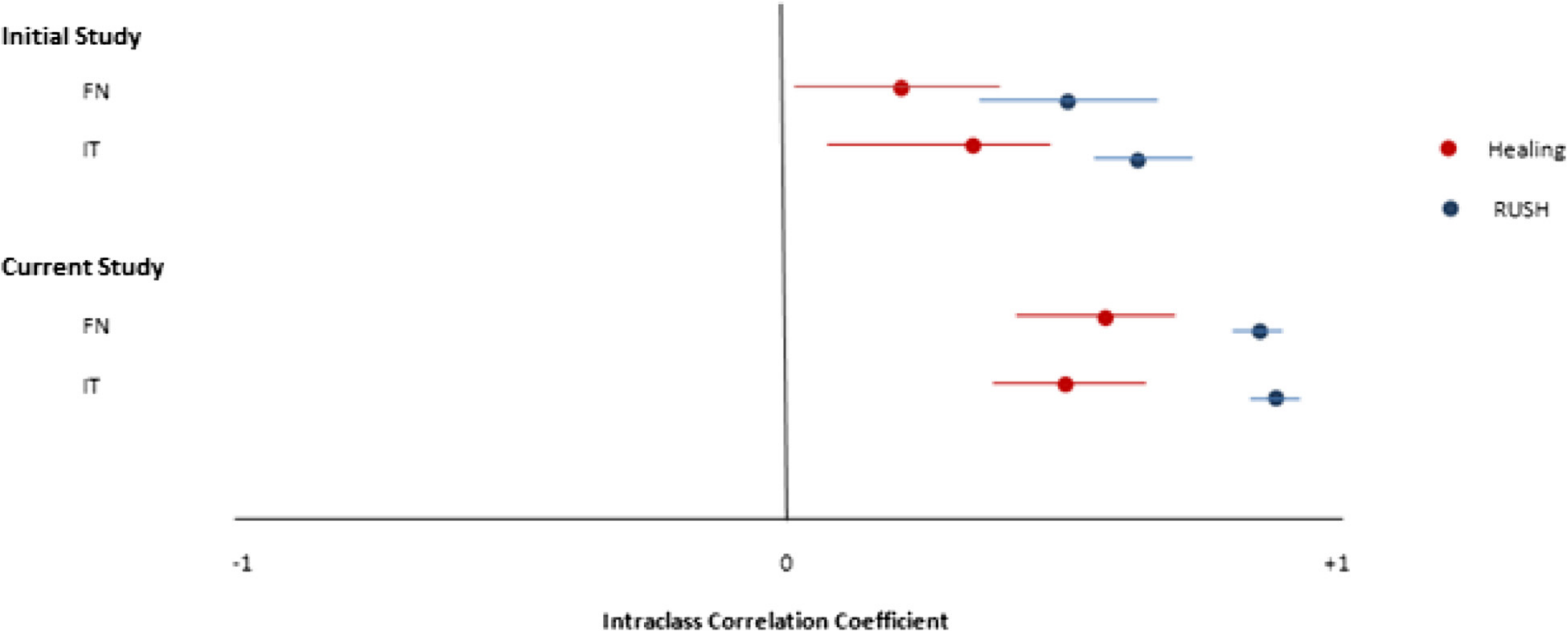
Reliability of healing and RUSH score for initial study (Single Radiographs) vs. current study (Serial Radiographs).

In the initial study completed assessing the RUSH checklist, reviewers were provided with a single radiograph for each case, and were unaware of when it was obtained with regard to surgery [11]. In the previous study, overall impression of fracture healing resulted in only fair agreement for both femoral neck (ICC = 0.22, 95% CI: 0.01-0.41) and intertrochanteric fractures (ICC = 0.34, 95% CI: 0.11-0.52). The comparison is shown in Figure 4 for both fracture types. The agreement for RUSH scores improved in the current study compared to the previous study [11].

### Correlation between the assessment of fracture healing and the RUSH score

**Figure 5:**
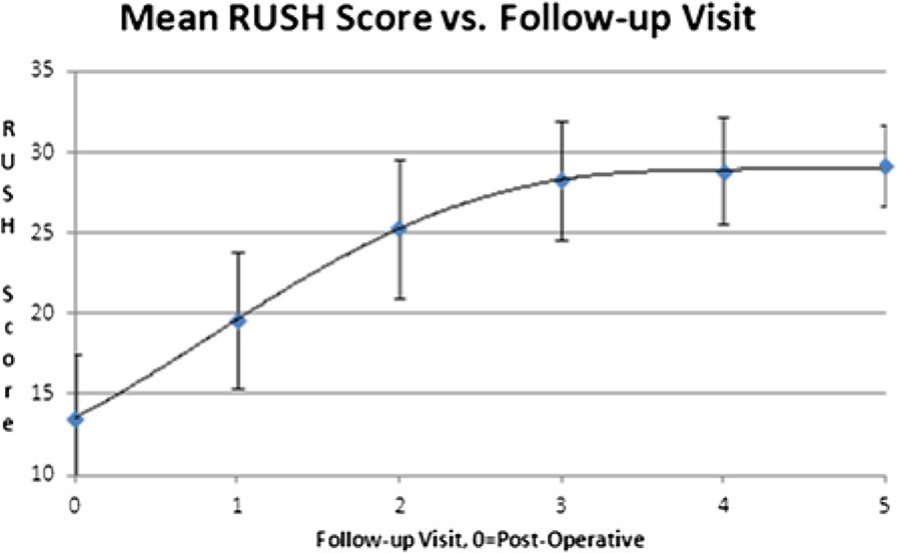
Changes in the Mean RUSH score with increasing time from baseline, femoral neck fractures.

**Figure 6:**
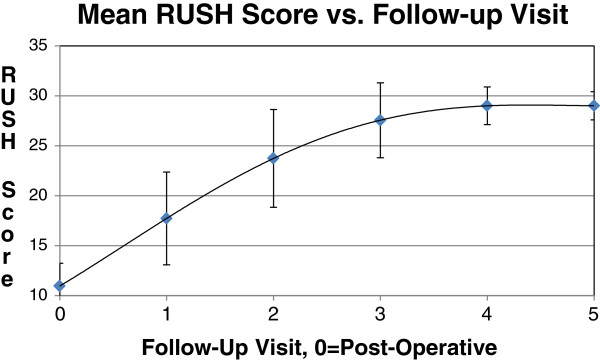
Changes in the Mean RUSH score with increasing time from baseline, intertrochanteric fractures.

A regression analysis was performed to determine the correlation between fracture healing and the calculated RUSH score. Receiver Operating Characteristic (ROC) analysis showed a high strength of association with an area under the curve of 0.993 for femoral neck cases and 0.989 for intertrochanteric cases. We additionally observed an asymptotic increase in the RUSH score toward the maximum score of 30 as the number of visits from the post-operative baseline increased. This is illustrated by Figure 5 for femoral neck fractures and Figure 6 for intertrochanteric fractures.

## Discussion

Our reliability study of 100 femoral neck and 100 intertrochanteric fracture cases with 6 reviewers identified three key findings: 1) inter-observer agreement on fracture healing is moderate between radiologists and orthopedic surgeons, 2) agreement is significantly improved to near perfect with the use of the RUSH checklist, and 3) agreement is significantly improved when using sequential radiographs compared to radiographs from a single, unknown time point.

As we expected, the introduction of serial radiographs in which the time from surgery was known significantly improved agreement between the reviewers for both the overall impression of fracture healing and the RUSH score. Perhaps more surprising and intriguing was the extent to which agreement between reviewers improved with the use of the RUSH checklist. This is suggestive that the RUSH checklist can be a useful clinical tool to assess hip fractures in a way that improves consistency and reliability between clinicians, as well as increasing the utility of hip fracture radiographs. This is promising given the need for a more standardized, objective manner of assessing the healing of hip fractures. This is illustrated by the fact that fracture healing is a frequent end point outcome in orthopedic research trials; therefore, differing and subjective accounts of fracture healing can dramatically affect the perceived efficacy of a treatment [14]. Many clinicians also base their treatment decisions on when a fracture is healed [14]. Discrepancies between interpretations of healing between radiologists and surgeons are also evidenced and can potentially lead to misunderstandings in a clinical setting [15,16].

With regard to the timing of the radiographs, there was generally less consensus between radiologists and surgeons for radiographs obtained earliest after surgery (0–3 months), and a higher degree of agreement for radiographs taken at a later time point (6 months or more after surgery). The exception to this is for the RUSH scores for intertrochanteric fractures, in which the agreement between groups decreased slightly for later time points. Interestingly, the agreement between groups was higher when the RUSH checklist was used at the earliest time points, from 0 to 3 months after surgery (ICC = 0.709 and 0.816 for femoral neck and intertrochanteric fractures, respectively), than for the overall impression of fracture healing at the latest time points, 6 or more months after surgery (ICC = 0.466 and 0.536 for femoral neck and intertrochanteric fractures, respectively). This suggests that the RUSH checklist greatly improves agreement and assessment of radiographs.

Tibial fractures, while distinct from the hip fractures that are the subject of this study, offer an interesting and important model in an attempt to standardize healing assessment. In light of studies showing poor agreement on tibial fracture healing, the Radiographic Union Score for Tibial fractures (RUST) score was developed as a means to improve the reliability of tibial healing [12,17,18]. As hoped, the RUST checklist did provide substantial and improved inter-rater agreement [12].

A review of the literature underscores the inconsistency of healing assessment as several studies point out the subjective nature of assessment and its possibly detrimental consequences in both the clinical and academic settings [10,19-22]. Davis et al. identify the importance of accurately defining union and notes the central role played by radiographs in the interpretation of fracture healing, despite the apparent difficulties with interpretation [14].

Other studies of interest to us are those that assess reviewer agreement on fracture classification systems using radiographs [23-25]. A test of the AO classification system using plain radiographs yielded poor agreement [23]. Eight observers assessing fractures radiographically using Garden’s classification system also had low agreement [24]. A study by Bjorgul et al., while not looking at classification systems, found only poor to moderate agreement when hip fracture radiographs were used to assess various radiographic signs considered to be predictive of healing abnormalities [26]. These all highlight the problems of radiographic interpretation in terms of inconsistency and the lack of reproducible results between clinicians. This makes our near perfect agreement for the RUSH checklist seem even more promising and significant in consideration of this information.

Our study specifically examines reliability of healing from a strictly imaging perspective, as the interpretation of radiographs is often central to the assessment of healing. However, there is also a diversity of opinion regarding the best method to determine the healing status of a fracture. The literature compares different methods of assessing healing, ranging from radiographic imaging, clinical assessment such as weight bearing pain, questionnaires, or a combination of these and other methods [27]. Indeed, there is evidence that the optimal method of assessing healing involves a combination of radiographs and clinical assessment, which is usually the case in the clinical setting [28,29]. This is support for additional studies in the future that investigate the impact on reliability from the inclusion of clinical information in addition to the radiographic imaging [29]. Still, radiographic imaging is a critical part of the assessment and it is therefore important to ensure reliability in interpretation.

There were several strengths to our study. The cases that we selected were diverse in terms of the nature of their operative treatment and the inclusion of both femoral neck and intertrochanteric fractures reflect the most common types of hip fractures encountered in practice. The large number of cases was also helpful in terms of ensuring our study had adequate power. The reviewers provided diverse perspectives due to the inclusion of both radiologists and orthopedic surgeons on the reviewer panel, while their high level of training and experience afforded expert clinical judgment. The use of Global Adjudicator™, an online adjudication system, helped to ensure the independence of reviews as the assessments were all completed remotely [30]. Using serial radiographs with the time from surgery known to the reviewers may also be seen as a strength of the study, as it is more reflective of actual clinical practice.

Conversely, some limitations of our study include the potentially limited applicability of assessment to other reviewers who may lack similar levels of training and especially experience. In a similar respect, our reviewers had the advantage of previously participating in a study similar to this one in which plain radiographs were also assessed for healing using the RUSH checklist. This gives the reviewers an additional level of comfort and experience with the RUSH checklist that others may not immediately possess. On the other hand, the positive aspect of this is that the results suggest that increased experience with the RUSH checklist improves performance and consistency. An additional limitation is that the RUSH checklist has not yet been validated, though this can be accomplished with further studies. As noted in the results, there is a high correlation between the fracture healing and the overall RUSH score, but the interpretation of this is limited by the knowledge that the reviewers assessed both variables simultaneously, as opposed to at two separate time points in time. Furthermore, in the collection of radiographs, the lateral images available were not always true views. The majority of the images obtained were cross-table lateral images; however, when this was not possible an oblique view was used. Although this led to images that were not always strictly comparable, these images are those that are typically seen in practice, adding to the generalizability of our results.

## Conclusions

We propose the RUSH checklist as a potential method of improving fracture healing agreement among clinicians based on the results from our study. The high level of agreement for the RUSH score seen in our results suggests that the RUSH checklist is a promising method of improving reliability and providing objectivity in the very subjective area of fracture healing assessment. There is a need for further studies evaluating the reliability and efficacy of RUSH checklist. Future research initiatives may include the evaluation of radiographs along with clinical notes to provide the information obtained from a clinical assessment for increased generalizability. Furthermore, the RUSH checklist should be evaluated for feasibility and validity of its implementation into clinical practice.

## Competing interests

The authors declare that they have no competing interests.

## Authors’ contributions

MB and SS conceived of the study and participated in its design and coordination, and drafting of the manuscript. MC, NP, HC, OA, RC, and BP performed the assessments and scoring of the radiographs and participated in the consensus meetings. SB performed the statistical analysis and assisted with draft the manuscript. AH participated in the study’s coordination, assisted with the statistical analysis and participated in editing the manuscript. All authors read and approved the final manuscript.

## Authors’ information

From the Assessment Group for Radiographic Evaluation and Evidence

(AGREE) Study Group*

McMaster University, Hamilton, Ontario

## Disclaimer

Funding for this research was provided a research grant from AMGEN Inc.

Dr. Bhandari was funded, in part, by a Canada Research Chair.

## Pre-publication history

The pre-publication history for this paper can be accessed here:

http://www.biomedcentral.com/1471-2474/14/70/prepub

## Supplementary Material

Additional file 1: Appendix A The RUSH Checklist. Click here for file
