# Supplementary figures and images for: Radiographic union score for hip substantially improves agreement between surgeons and radiologists
Source: BMC Musculoskelet Disord. 2013 Feb 25;14:70. doi: 10.1186/1471-2474-14-70 (PMC3599458; doi:10.1186/1471-2474-14-70)

**APPENDIX A**

**
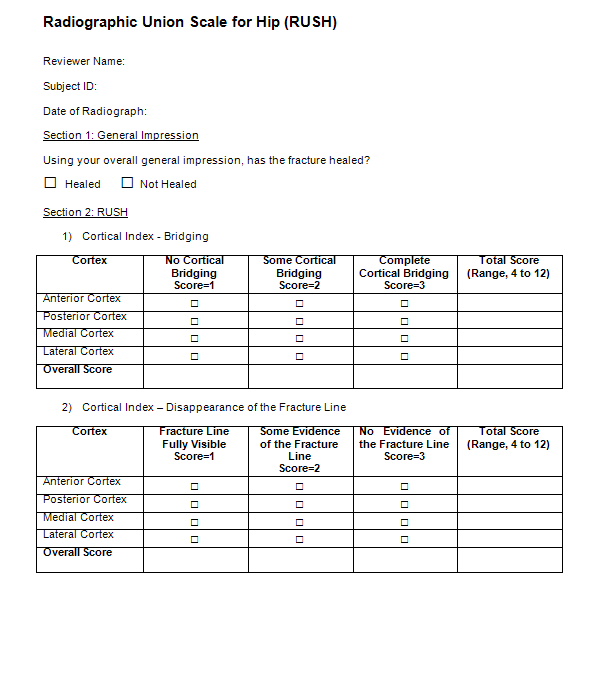
**

**
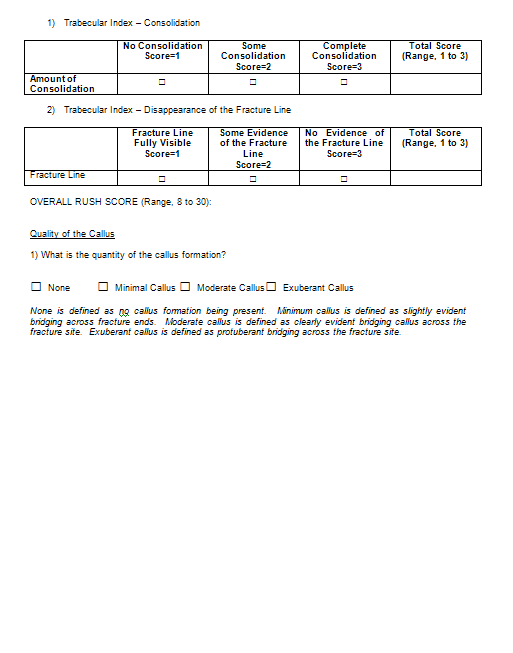
**

Supplement: Additional file 1: Appendix A — The RUSH Checklist. [file 1471-2474-14-70-S1.docx]
